# Soluble γc cytokine receptor suppresses IL-15 signaling and impairs *i*NKT cell development in the thymus

**DOI:** 10.1038/srep36962

**Published:** 2016-11-11

**Authors:** Joo-Young Park, Yuna Jo, Eunhee Ko, Megan A. Luckey, Yoo Kyoung Park, Se-Ho Park, Jung-Hyun Park, Changwan Hong

**Affiliations:** 1 Experimental Immunology Branch, National Cancer Institute, NIH, Bethesda, MD 20892, USA; 2 Department of Anatomy, Pusan National University School of Medicine, Yangsan 626-870, South-Korea; 3 Department of Medical Nutrition, Graduate School of East-West Medical Science, Kyung Hee University, Yongin 446-701, South-Korea; 4 School of Life Sciences and Biotechnology, Korea University, Seoul 136-701, South-Korea

## Abstract

The soluble γc protein (sγc) is a naturally occurring splice isoform of the γc cytokine receptor that is produced by activated T cells and inhibits γc cytokine signaling. Here we show that sγc expression is also highly upregulated in immature CD4^+^CD8^+^ thymocytes but then downregulated in mature thymocytes. These results indicate a developmentally controlled mechanism for sγc expression and suggest a potential role for sγc in regulating T cell development in the thymus. Indeed, sγc overexpression resulted in significantly reduced thymocyte numbers and diminished expansion of immature thymocytes, concordant to its role in suppressing signaling by IL-7, a critical γc cytokine in early thymopoiesis. Notably, sγc overexpression also impaired generation of *i*NKT cells, resulting in reduced *i*NKT cell percentages and numbers in the thymus. *i*NKT cell development requires IL-15, and we found that sγc interfered with IL-15 signaling to suppress *i*NKT cell generation in the thymus. Thus, sγc represents a new mechanism to control cytokine availability during T cell development that constrains mature T cell production and specifically *i*NKT cell generation in the thymus.

Cytokines of the γc family play critical roles in T cell development in the thymus^1^,^2^. Among others, IL-7 is essential for thymopoiesis^2^,^3^, IL-2 is necessary for Foxp3^+^ Treg cell development^4^,^5^, and IL-15 is required for the development of invariant NKT (*i*NKT) cells in the thymus^6^,^7^. Notably, γc cytokine responsiveness is mostly acquired during or after initiation of lineage-specification during thymocyte development. As such, pre-selection CD4^+^CD8^+^ double-positive (DP) thymocytes are unresponsive to IL-7^8^,^9^, and IL-7 responsiveness is acquired upon cessation of positive selection signals in post-selection CD4 or CD8 single-positive (SP) thymocytes^3^,^10^,^11^. DP thymocytes are also unresponsive to IL-2, and IL-2 responsiveness in CD4 thymocytes is only acquired by strong TCR engagements that also upregulate expression of the transcription factor Foxp3^12^,^13^. We and others have previously proposed that such γc unresponsiveness in DP thymocytes is achieved through multiple redundant mechanisms^9^,^10^,^14^, and that prevention of pro-survival γc cytokine signaling is critical to ensure selection of self-peptide/MHC-specific, immunocompetent T cells^10^,^15^,^16^.

The ability to respond to a specific γc cytokine depends on surface cytokine receptor expression. IL-7Rα expression is silenced in pre-selection DP thymocytes but induced upon TCR-mediated positive selection, which correlates with the inability of IL-7 signaling by DP cells^17^,^18^,^19^. Moreover, IL-2 receptor expression is absent in DP and most CD4SP thymocytes, but upregulated in Foxp3^+^ Treg precursor cells which depend on IL-2 for survival^13^. IL-2 receptor expression is also critical for generation of *i*NKT cells who utilize IL-2Rβ to be signaled by IL-15, a critical survival and differentiation cytokine for *i*NKT cells^7^,^20^,^21^. The failure to express cytokine receptors in a stage-specific manner is detrimental for thymocyte development and lineage differentiation^22^,^23^. Thus, understanding the molecular mechanisms that control expression of γc family cytokine receptors during differentiation of distinct thymocyte subsets is an important issue in T cell biology. Interestingly, and in contrast to the cytokine-proprietary receptors, the regulatory mechanism of γc expression has remained largely unmapped. γc is expressed on immature CD4, CD8 double-negative (DN) thymocytes for survival and proliferation, and γc expression is also upregulated upon positive selection to mediate lineage choice and effector cell differentiation^19^. Importantly, γc expression is downregulated on immature DP cells, presumably to suppress aberrant γc cytokine signaling that could provide pro-survival effects on pre-selection thymocytes^19^. However, the molecular pathway that suppresses γc expression on DP cells remains still veiled.

We have previously identified alternative splicing of γc pre-mRNA as a new mechanism to reduce surface γc protein expression^24^. The γc gene is encoded in 8 exons, and exon 6 encodes the entire transmembrane domain^25^. While the full-length γc protein is a transmembrane protein, the new splice isoform lacks exon 6 and thus the transmembrane region, making it a soluble secreted protein. Because the soluble form of γc (sγc) is generated at the expense of membrane γc protein expression, sγc expression inversely correlates with the amount of surface γc expression. Therefore, sγc expression represents a novel mechanism to suppress γc expression on cell surface.

In the present study, we now identify DP thymocytes as a major source of sγc and we propose that alternative splicing into sγc could promote establishing the low level of surface γc on pre-selection DP thymocytes. Moreover, because sγc proteins suppress signaling of γc cytokines, such as IL-2 and IL-7^24^, sγc production by DP thymocytes would create an overall suppressive milieu for γc cytokine signaling in the thymus. In fact, we found that sγc overexpression resulted in significantly diminished percentages and numbers of thymic *i*NKT cells, which are critically dependent on IL-15 signaling for their development and differentiation^6^,^7^,^20^,^21^. Specifically, increased sγc expression resulted in the loss of HSA^lo^ mature *i*NKT cells, as it interfered with upregulation of anti-apoptotic Bcl-2 expression and induced increased cell death. Collectively, these data demonstrate a previously unappreciated role for sγc in downregulating surface γc expression and also in dampening γc cytokine signaling in thymocytes, which can inhibit the generation and differentiation of specific T cell subsets in the thymus.

## Results

### γc family cytokine receptor expression on thymocytes

**Figure 1:**
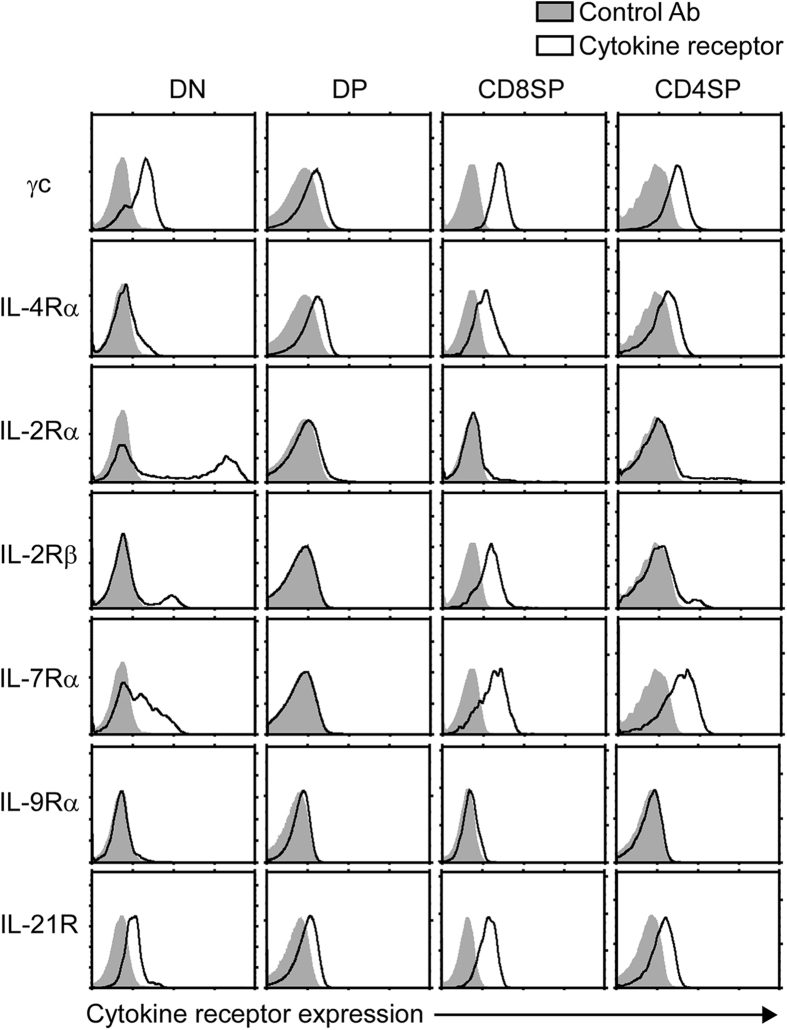
Cell surface expression of γc family cytokine receptors on thymocytes. Expression of the indicated cytokine receptors were assessed on thymocyte subpopulations. Data are representative of 5 independent experiments.

Surface staining for γc family cytokine receptors revealed distinct and stage-specific expression of individual cytokine receptors (Fig. 1). Most γc family cytokine receptors were found on both CD4 and CD8 single positive (SP) thymocytes but absent on immature DP thymocytes. IL-4Rα, IL-21R and γc differed as they were also expressed on DP cells. Consequently, DP thymocytes would be unable to respond to IL-7, but they are equipped with IL-4 and IL-21 responsiveness. Importantly, while DP cells did express γc, the amount of surface γc was markedly lower compared to that on immature DN or mature SP thymocytes (Fig. 1). These results indicated and confirmed that γc expression is a developmentally controlled event that is specifically suppressed on pre-selection DP cells^19^. Reduced γc expression presumably helps avoiding signaling by pro-survival γc cytokines which could interfere with TCR-induced positive selection as previously suggested^16^.

**Figure 2:**
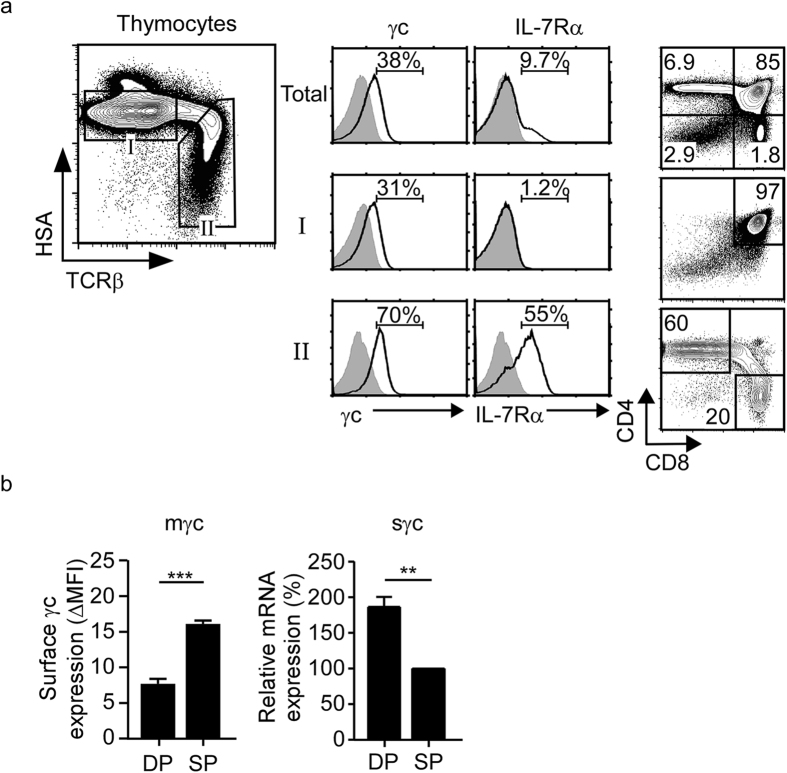
Immature DP thymocytes express low levels of membrane γc but high levels of soluble γc proteins. (**a**) Stage-specific expression of cell surface IL-7Rα and γc during thymocyte development. Contour plot shows HSA and TCRβ staining on total thymocytes (left). Discrete developmental stages were defined by surface HSA and TCRβ expression. IL-7Rα and γc expression (open histograms) were assessed on gated thymocytes and overlaid with control antibody staining (shaded histogram) (middle). Contour plots show CD4/CD8 profiles of gated thymocyte populations (right). Data are representative of 6 independent experiments. (**b**) γc protein and mRNA expression in pre-selection DP and post-selection SP thymocytes. γc surface protein levels on gated DP (CD4^+^CD8^+^) and SP (TCRβ^+^HSA^lo^) thymocytes (left). Relative expression of sγc mRNA in sorted DP and SP thymocytes determined by qRT-PCR (right). Data are the mean and SEM of 4 independent experiments.

To correlate γc expression with positive selection, next, we analyzed surface expression of γc and IL-7Rα on HSA^hi^TCRβ^lo^ pre-selection (gate I) and HSA^lo^TCRβ^hi^ post-selection thymocytes (gate II) (Fig. 2a). Expression of IL-7Rα and γc was low on gate I immature DP thymocytes but upregulated on gate II mature SP cells, which illustrated developmental control of cytokine receptor expression in thymocytes. The molecular mechanism that downregulates γc expression on DP thymocytes is not known. However, we previously reported a post-transcriptional mechanism that can downregulate surface γc expression^24^. Specifically, we found that alternative splicing of γc transcripts produced a soluble form of γc (sγc) that was generated at the expense of membrane γc (mγc) protein expression^24^. Thus, increase in sγc expression conversely results in reduced surface γc expression. Interestingly, here we found that DP thymocytes expressed markedly higher levels of sγc transcripts than mature SP thymocytes (Fig. 2b right), and that increased sγc expression inversely correlated with decreased mγc protein expression in the same cells (Fig. 2b). These data suggest that alternative splicing of γc mRNA might contribute to downregulation of surface γc expression on DP thymocytes. Moreover, DP cells comprise up to 90% of total thymocytes so that they are a major source of sγc proteins, and thus render the thymus into an sγc-rich environment. However, if sγc plays a role in thymocyte differentiation is not known.

### sγc overexpression impairs thymocyte development

**Figure 3:**
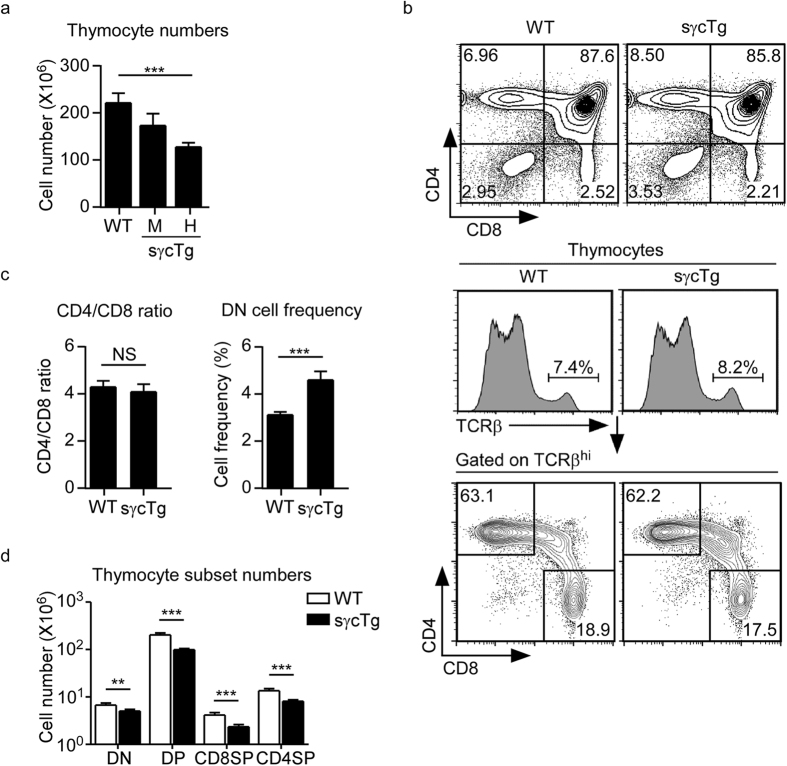
Thymocyte development and differentiation in sγc-transgenic mice. (**a**) Total thymocyte numbers in sγcTg and WT control mice. M, sγc medium expresser; H, sγc high expresser. Results are the mean and SEM of 12 independent experiments. (**b**) CD4 versus CD8 profile of total thymocytes (top) and TCRβ^hi^ gated thymocytes (bottom) in WT and sγcTg mice. (**c**) CD4/CD8 ratio of mature SP thymocytes (left) and frequency of DN thymocytes (right) in WT and sγcTg mice. (**d**) Cell numbers of thymocyte subsets in WT and sγcTg mice. Data are summary of 8 independent experiments with each 10 WT and 15 sγcTg mice.

To interrogate sγc’s effect on T cell development, we analyzed thymocytes in sγc transgenic mice (sγcTg)^24^. To generate sγcTg mice, a murine sγc cDNA was placed under the control of a human CD2 mini-cassette so that sγc is overexpressed in all T lineage cells. Increased sγc expression significantly reduced total thymocyte numbers, and we observed an inverse correlation of sγc expression and total thymocyte numbers in WT, sγc medium (M) and sγc high (H) expresser transgenes (Fig. 3a). All further experiments in this study were done with the sγc high expresser line. Assessing thymocyte profiles of sγcTg mice did not reveal any significant changes in TCRβ^hi^ mature T cell generation (Fig. 3b) or in CD4/CD8 lineage commitment (Fig. 3c left). However, we did find a significant increase in DN cell frequency (Fig. 3c right), suggesting a developmental defect in DN to DP cell transition, which would also explain the reduction in thymocyte numbers in sγcTg mice (Fig. 3d)^26^.

**Figure 4:**
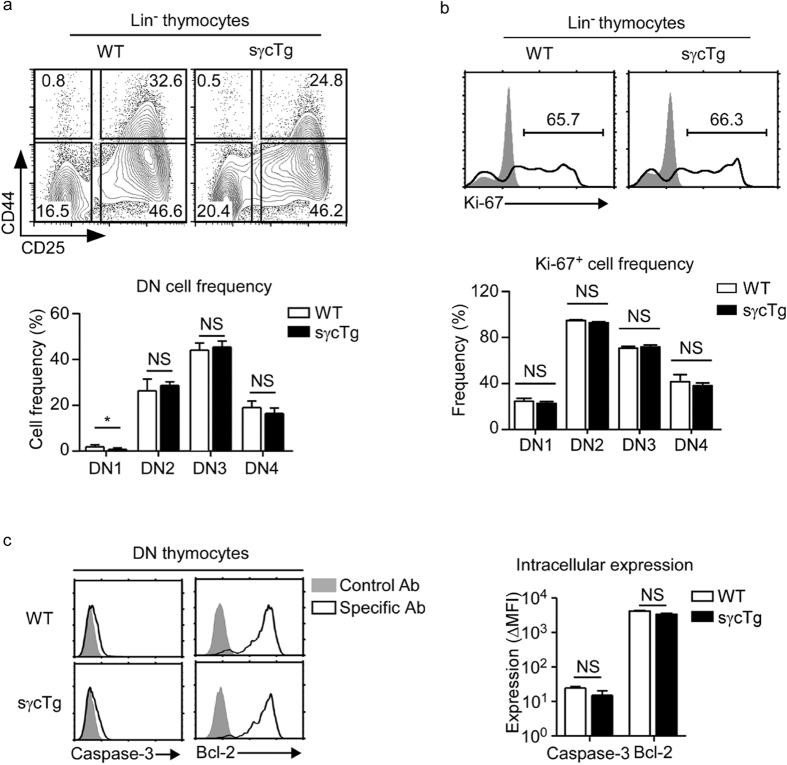
DN stages differentiation in WT and sγcTg mice. (**a**) DN1-DN4 differentiation in lineage marker negative (Lin^–^) DN thymocytes of WT and sγcTg mice (top). Bar graph shows frequencies of individual DN subsets (bottom). Data are the summary of 3 independent experiments with a total of 5 WT and 8 sγcTg mice. (**b**) Intracellular Ki-67 staining of Lin^-^ DN thymocytes in WT and sγcTg mice (top). Bar graph shows Ki-67^+^ frequencies in individual DN subsets (bottom). Data are the summary from each two WT and sγcTg mice. (**c**) Caspase-3 activity and intracellular Bcl-2 expression were determined by flow cytometry in DN thymocytes of WT and sγcTg mice (left). Bar graphs show mean and SEM of each 3 WT and sγcTg mice (right).

To directly address this point, we examined surface CD44 and CD25 expression in lineage marker negative DN thymocytes and determined DN1-DN4 differentiation in sγcTg and WT thymocytes (Fig. 4a)^27^. Contrary to our expectation, however, we did not find any significant differences in DN1–4 subset frequencies between WT and sγcTg mice. We also did not find any significant difference in Ki-67 expression in individual DN subsets (Fig. 4b), suggesting that the proliferative potential of sγcTg DN cells did not differ from WT thymocytes. Finally, to examine the possibility that increased cell death of DN thymocytes would account for reduced cell numbers, we assessed caspase-3 activity and intracellular Bcl-2 contents in sγcTg DN thymocytes (Fig. 4c). Decreased Bcl-2 expression is associated with increased susceptibility to apoptosis, and elevated caspase-3 activity is indicative of increased cell death^28^,^29^. However, we did not find any differences in their expression either between sγcTg and WT DN thymocytes (Fig. 4c).

Notably, DP thymocytes in sγcTg mice had been previously reported to contain increased percentages of CD25-positive cells^24^. Also, surface CD25 expression is diluted during the proliferative burst of DN to DP transition^30^. Thus, these results collectively suggested that reduced thymocyte numbers and increased DN cell percentages are results of reduced cell proliferation during DN to DP cell transition and not due to a developmental arrest at DN2/DN3 stage of T cell development.

### Thymic development of γδ T cells and Foxp3^+^ Treg cells in sγcTg mice

**Figure 5:**
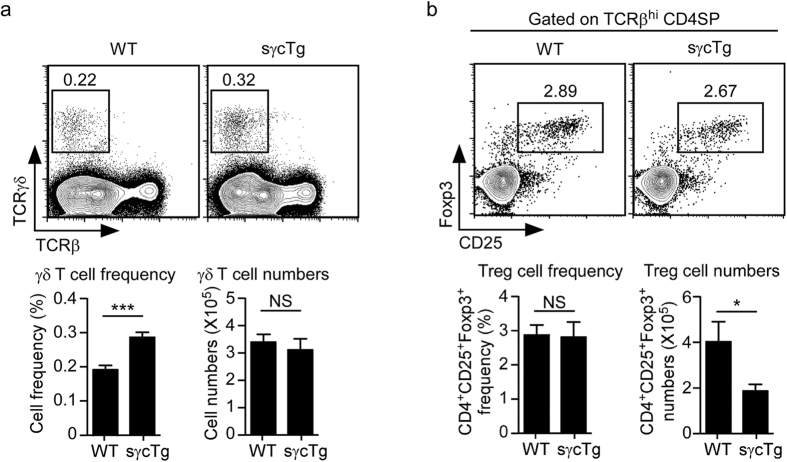
Thymic γδ T cell and Foxp3^+^ Treg cell generation are unaffected in sγcTg mice. (**a**) Frequency and number of γδ T cells were determined from WT and sγcTg thymocytes. Data are the mean and SEM of 11 sγcTg and WT control thymocytes. (**b**) Frequency and number of Foxp3^+^ Treg cells were determined from WT and sγcTg mice. Data are the mean and SEM of 11 sγcTg and WT control thymocytes.

To further assess the impact of increased sγc expression, next, we analyzed generation of individual thymic T cell subsets. We first assessed γδ T cell generation in the thymus and found it unaffected in sγcTg mice. Thymic γδ T cell numbers did not differ between WT and sγcTg mice, and because overall thymocyte numbers were decreased in sγcTg mice, this translated into increased percentages of γδ T cells in the thymus (Fig. 5a). Next, we examined generation of Foxp3^+^ T regulatory (Treg) cells in sγcTg thymocytes, and found a significant decrease in Foxp3^+^CD25^+^ CD4SP Treg cell numbers (Fig. 5b). However, we did not find a decrease in Foxp3^+^CD25^+^ cell percentages among CD4SP thymocytes (Fig. 5b), which indicated that reduced Foxp3^+^ Treg cell number is due to an overall impairment in thymopoiesis and not because of a specific defect in thymic Treg cell generation.

### sγc overexpression impairs *i*NKT cell generation

**Figure 6:**
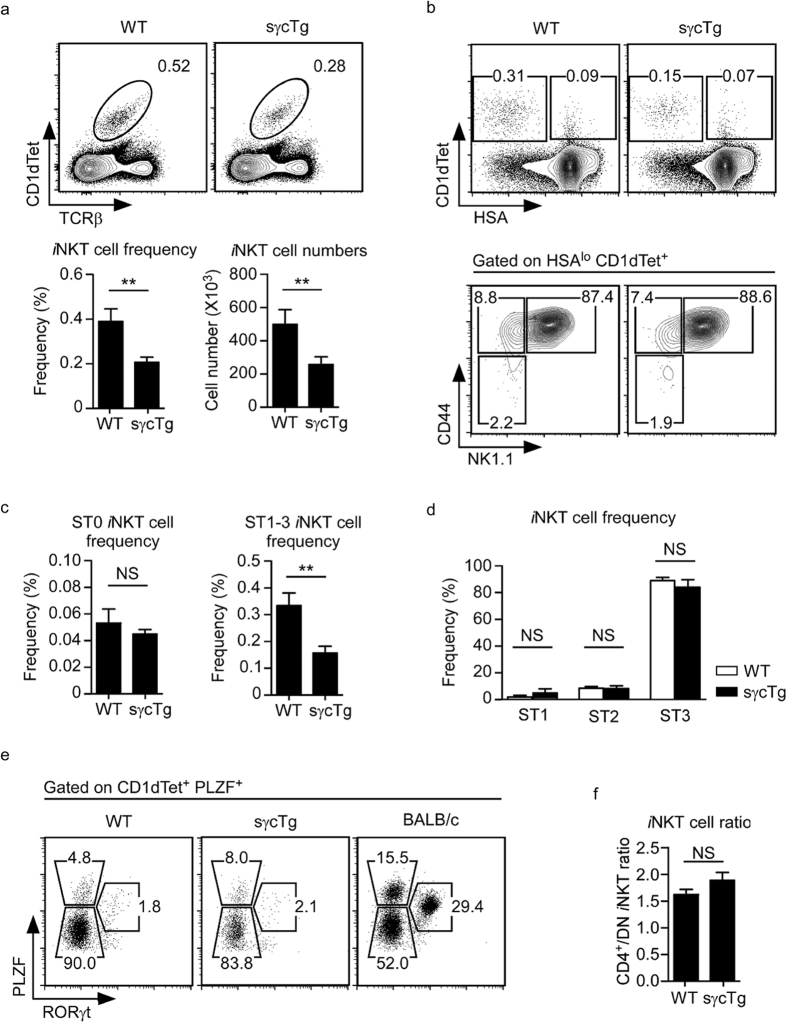
Impaired generation of thymic *i*NKT cells in sγcTg mice. (**a**) Frequency and number of *i*NKT cells in WT and sγcTg thymocytes. Data show summary (mean ± SEM) from 13 sγcTg and 9 WT control thymocytes. (**b**) *i*NKT cell stages in WT and sγcTg thymic *i*NKT cells. CD1dTet^+^ HSA^lo^ mature *i*NKT cells (top) were assessed for CD44 and NK1.1 expression (bottom). Results are representative of 13 sγcTg and 9 WT control mice in 4 independent experiments. (**c**) Frequencies of immature Stage 0 (ST0) and mature stage 1–3 (ST1–3) *i*NKT cells in WT and sγcTg mice. Data show mean and SEM of 13 sγcTg and 9 WT control thymocytes. (**d**) Frequencies of distinct *i*NKT cell stages in WT and sγcTg thymocytes. Data show mean and SEM of 13 sγcTg and 9 WT control thymocytes. (**e**) Transcription factor expression in thymic *i*NKT cells from WT (C57BL/6), sγcTg, and BALB/c mice as assessed by intracellular staining for PLZF versus RORγt. Numbers indicate percentages of PLZF^lo^RORγt^−^ (NKT1) cells, PLZF^hi^RORγt^−^ (NKT2) cells and PLZF^int^RORγt^+^ (NKT17) cells among CD1dTet^+^ *i*NKT cells that expressed PLZF. (**f**) CD4^+^ versus DN *i*NKT cell ratio in WT and sγcTg thymocytes. Data show mean and SEM of 3 independent experiments with each 3 WT and 4 sγcTg mice.

*i*NKT cells are thymus-generated innate T lineage cells that depend on IL-15 for their development and differentiation^7^,^31^,^32^. *i*NKT cells can be identified by their TCR reactivity to lipid-loaded CD1d tetramers (CD1dTet)^33^, and here we found that both frequency and number of CD1dTet^+^ *i*NKT cells were significantly reduced in sγcTg thymocytes (Fig. 6a). Conventionally, *i*NKT cell development had been understood based on cell surface HSA (CD24), CD44 and NK1.1 expression^34^,^35^,^36^. The most immature CD1dTet^+^ *i*NKT cells express high levels of HSA and are defined as stage 0 *i*NKT cells. Upon further maturation, *i*NKT cells lose HSA expression but start expressing CD44 and then NK1.1, so that CD44^–^NK1.1^−^ cells are stage 1, CD44^+^NK1.1^−^ cells are stage 2, and CD44^+^NK1.1^+^ cells are referred to as stage 3 *i*NKT cells^35^. Assessing WT and sγcTg thymic *i*NKT cells revealed no significant differences between WT and sγcTg mice when comparing in individual stages (Fig. 6b–d). However, there was a significant loss of sγcTg *i*NKT cells when comparing the combined frequency of mature *i*NKT cells, *i.e.* stage 1–3 (Fig. 6c). Because the frequency of immature stage 0 *i*NKT cells did not differ between sγcTg and WT control mice, these results suggest that sγc overexpression did not target a specific developmental stage but rather induces an overall reduction of thymic *i*NKT cells.

*i*NKT cells can be also categorized into discrete subsets based on their function and transcription factor expression^37^. PLZF^lo^ T-bet^+^ cells correspond to IFNγ-producing NKT1, PLZF^hi^RORγt^−^ cells are IL-4-producing NKT2, and PLZF^int^RORγt^+^ are IL-17-producing NKT17 cells^38^. In C57BL/6 (B6) WT mice, the majority of thymic *i*NKT cells are NKT1 cells with only few NKT2 and NKT17 cells. Such *i*NKT cell distribution is not developmentally fixed, and changes with mouse strains as illustrated by significantly increased NKT2 and NKT17 cell percentages in BALB/c mice (Fig. 6e)^37^. We found that sγcTg mice, which were maintained on a B6 background, showed identical distribution of NKT subsets to control WT B6 cells (Fig. 6e). Additionally, when dividing *i*NKT cells into two major subsets of CD4^+^ and DN *i*NKT cells^32^, we also did not find any difference between sγcTg and WT mice (Fig. 6f). Collectively, these results demonstrate that sγc overexpression is detrimental for thymic *i*NKT cell generation, and that sγc affected *i*NKT cell frequency and number without targeting a specific *i*NKT subset or specific developmental stage.

### sγc interferes with IL-15 signaling in *i*NKT cells

**Figure 7:**
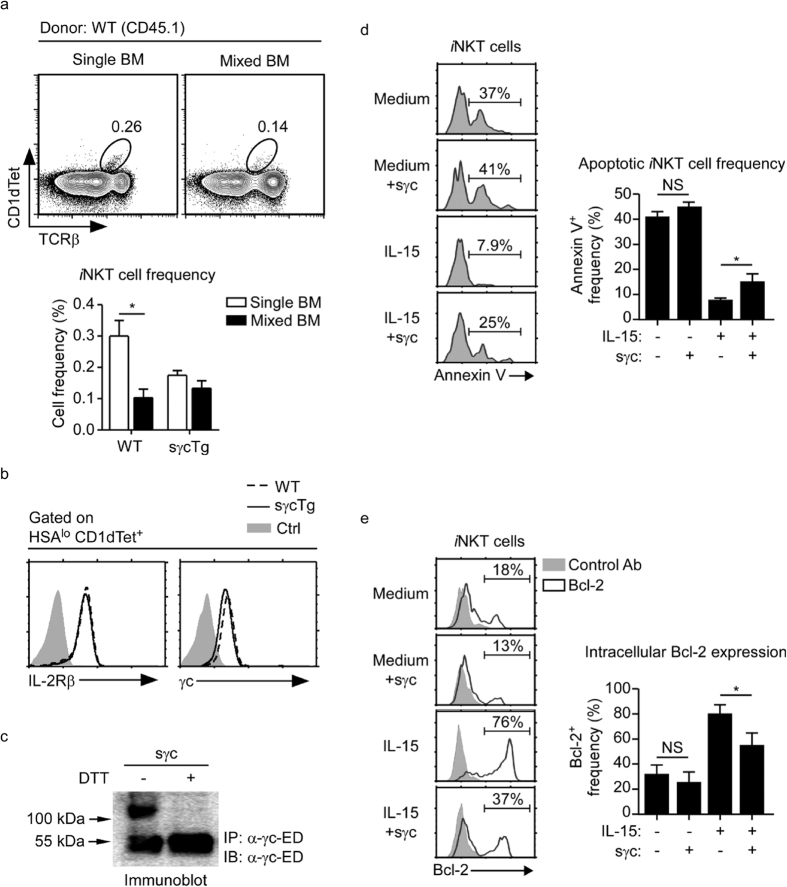
Recombinant sγc proteins suppress IL-15 signaling. (**a**) *i*NKT cells in thymus of bone marrow chimeric mice. Bone marrow of WT (CD45.1) and sγcTg (CD45.2) mice were transferred into irradiated RAG-deficient mice, and thymocytes were analyzed 8 weeks after reconstitution. *i*NKT cell generation was assessed in WT-origin donor cells in single WT (single BM) or unequally-mixed (1:2 ratio of WT versus sγcTg, mixed BM) bone marrow chimeric mice. Bar graphs show percentages of *i*NKT cells among CD45.1^+^ or CD45.2^+^ thymocytes in single BM and mixed BM mice. Data are representative and summary of 3 independent experiments. (**b**) Surface IL-2Rβ and γc expression on mature HSA^lo^CD1dTet^+^-gated WT and sγcTg *i*NKT cells. Results are representative of 6 sγcTg and 4 WT control mice from 2 independent experiments. (**c**) Expression of recombinant sγc proteins. Culture supernatant of sγc expressing 293 T cells were immunoprecipitated (IP) and immunoblotted (IB) for sγc proteins using anti-γc ectodomain antibodies (α-γc-ED). Immunoprecipitates were resolved by SDS-PAGE under reducing (+ DTT) or non-reducing conditions. (**d**) Thymic *i*NKT cell survival upon 3-day *in vitro* IL-15 stimulation in the presence or absence of recombinant sγc proteins. Cell viability was determined by Annexin V staining. Histograms are representative of 3 independent experiments (left). Bar graphs show mean and SEM of 3 independent experiments (right). (**e**) Intracellular Bcl-2 expression in thymic *i*NKT cells stimulated for 3 days with IL-15 in the presence or absence of recombinant sγc proteins. Histograms show representative results from three independent experiments (left). Bar graphs show mean and SEM of 3 independent experiments (right).

To further understand the molecular basis of *i*NKT cell loss in sγcTg mice, next we examined whether increased sγc expression is a cell intrinsic requirement to suppress *i*NKT cell generation. We generated bone marrow (BM) chimeras where WT origin donor cells were used to reconstitute thymus development in RAG-deficient host mice, either alone or mixed at an unequal ratio (1:2) with sγcTg origin bone marrow cells. When analyzing the frequency of WT donor origin (CD45.1) *i*NKT cells, we found that WT origin BM cells gave rise to significantly reduced frequencies of *i*NKT cells, if they developed in a mixed thymic environment with sγcTg origin thymocytes. Thus, sγcTg origin BM cells impaired the generation of *i*NKT cells not only for sγcTg but also for WT *i*NKT cells (Fig. 7a). These results indicate that sγc’s effect to suppress *i*NKT cell development is mediated by a cell extrinsic mechanism.

*i*NKT cell development in the thymus depends on IL-15^7^,^20^,^21^,^39^, and defect in *i*NKT cell generation in sγcTg mice could be a direct consequence of impaired IL-15 signaling. Thus, we assessed expression of surface γc and IL-2Rβ which are the signaling units of a functional IL-15 receptor^40^. We did not find any significant difference in γc and IL-2Rβ expression between WT and sγcTg *i*NKT cells, which indicated that sγcTg did not impair *i*NKT cell generation because of defects in cytokine receptor expression (Fig. 7b). To further examine if sγc protein interferes with IL-15 signaling, we examined IL-15 downstream signaling in *i*NKT cells in the presence or absence of recombinant sγc proteins. Recombinant sγc proteins were produced in 293 T cells, and we confirmed successful formation of disulfide-linked sγc homo-dimers which represent the bioactive form of sγc protein (Fig. 7c)^24^.

IL-15 signaling is considered critical for in *i*NKT cells because it induces expression of anti-apoptotic proteins^6^. Bcl-2 is a pro-survival factor downstream of IL-15 signaling, and we found that IL-15-induced Bcl-2 expression was profoundly impaired in the presence sγc proteins. In particular, recombinant sγc interfered with the pro-survival effect of IL-15 during *in vitro* culture of thymic *i*NKT cells, as illustrated by significantly increased Annexin V binding (Fig. 7d) and diminished Bcl-2 expression (Fig. 7e). Thus, sγc inhibits *i*NKT cell development in the thymus, presumably by inhibiting IL-15 signaling.

## Discussion

Generation of soluble γc cytokine receptors through alternative pre-mRNA splicing results in two distinct but interlaced events: production of sγc proteins and diminished surface γc protein expression^24^. Both events are detrimental for γc cytokine signaling. Notably, the effect of alternative splicing is limited to sγc producing cells themselves, but secretion of sγc proteins can influence the function of other cells *in trans*. Thus, the physiological role of sγc proteins can be wide-ranging and diverse. Here we assessed the effect of sγc expression on thymic development, and we show that increased sγc production results in impaired thymopoiesis, which is a process dependent on IL-7 signaling^3^, and also in diminished *i*NKT cell generation, which is an event dependent on IL-15 signaling^7^,^21^,^39^. Generation of IL-2-dependent Foxp3^+^ Treg cells or IL-7-dependent CD8SP thymocytes^5^,^10^,^41^, on the other hand, were not affected. These results propose a hierarchy in γc cytokine responsiveness of post-selection thymocytes, with IL-15 being highly susceptible to increased concentrations of inhibitory sγc proteins, and IL-2 and IL-7 signaling more resistant to sγc-mediated inhibition. Collectively, this study reports a new role for sγc in suppressing IL-15 signaling, and it demonstrates that sγc can affect generation and differentiation of mature T cell subsets in the thymus.

Because sγc is highly expressed by DP thymocytes and because DP thymocytes comprise the vast majority (~85%) of thymocytes^42^, these results further suggest a role for DP thymocytes as a major source of sγc protein that dampens γc cytokine signaling in the thymus. Consequently, secretion of sγc proteins represents a new function for DP cells, and it suggests that DP thymocytes play an active role in thymic T cell differentiation by modulating γc cytokine signaling. Conventionally, DP thymocytes have been considered as only a transient developmental stage that is short-lived and that serves no other purpose than providing a pool of random TCR repertoire to be positively selected by the thymic self-peptide/MHC complexes^43^,^44^. In fact, DP thymocytes do not produce cytokines, and they are not considered to participate in T cell selection or maturation. Moreover, DP thymocytes are metabolically inactive and do not consume nutrients or compete for pro-survival factors^41^. Along these lines, termination of IL-7Rα expression on DP thymocytes has been suggested to prevent DP cells from consuming IL-7 which would interfere with IL-7-dependent proliferation of DN thymocytes^18^,^23^. Thus, DP thymocytes are thought to be a developmentally inert population that do not affect or control differentiation or selection of T cells in the thymus. On the other hand, there is an increasing body of evidence that shows DP thymocytes actively participating in T cell development in a cell extrinsic fashion. Such an idea is illustrated by the requirement for DP thymocytes to promote γδ T cell signature gene expression in immature DN thymocytes^45^, and also by a requirement for SLAM-SLAM homotypic interactions among DP thymocytes for positive selection of *i*NKT cells^46^,^47^. In the current study, we report a new mechanism of how DP cells affect thymic T cell differentiation, which is through the secretion of inhibitory sγc proteins. We think that sγc is the first of a class of soluble factors that are expressed by DP cells to interfere with thymic development. Sγc differs from other factors expressed by DP thymocytes, such as lymphotoxin and SLAM^46^,^47^, because it is not expressed in a membrane-bound form and does not require cell-cell contact. Collectively, DP thymocytes are a major source of sγc proteins, and sγc sets the threshold for γc cytokine signaling and tunes γc cytokine responsiveness during T cell development in the thymus.

The inhibitory mechanism of sγc proteins has been previously described^24^. In brief, sγc proteins form homo-dimers that bind with high affinity to unliganded cytokine receptors, such as IL-7Rα and IL-2Rβ. Direct binding of sγc to IL-7Rα or IL-2Rβ sequesters these receptors and can prevent them from binding to membrane γc proteins, which is necessary for cytokine signaling. Because IL-2 and IL-15 share the same IL-2Rβ/γc complex for ligand binding and signaling^1^, by implication, sγc binding to IL-2Rβ should interfere with both IL-2 and IL-15 signaling. Interestingly, during sγcTg T cell development, we found that IL-15 but not IL-2-dependent events were impaired. *i*NKT cell development was significantly blunted but Foxp3^+^ Treg cell generation remained intact. These results suggested distinct susceptibility of IL-2 versus IL-15 signaling to sγc-mediated inhibition. Why IL-15 signaling would be more perceptive to sγc blockade than IL-2 signaling is not clear. As a potential explanation, we considered the fact that IL-15 signaling requires IL-15 trans-presentation by IL-15Rα^48^, and that *i*NKT cell development depends on IL-15Rα-mediated IL-15 trans-presentation by thymic stromal cells^49^,^50^. Formation of a quaternary complex of IL-2Rβ/γc hetero-dimers on one cell with an IL-15/IL-15Rα complex on another cell could be more susceptible to steric hindrance by sγc proteins than the assembly of a functional IL-2Rα, β/γc signaling complex on the same cell. Altogether, the current results demonstrate an interference of sγc with IL-15-dependent steps during T cell development, and confirm the *in vivo* significance of sγc proteins in thymocyte differentiation.

The roles of γc cytokines in thymocyte development are well appreciated. Positive selection and lineage choice are two distinct events^51^. While TCR signaling controls positive selection, γc signaling plays a critical role in lineage fate decision and differentiation of post-selection thymocytes^52^. Following positive selection, IL-7 signaling induces Runx3 expression and imposes CD8 lineage choice^10^,^16^,^19^, whereas IL-2 signaling is necessary to upregulate Foxp3 and promote Treg cell differentiation in CD4SP cells^5^. For *i*NKT cells, IL-15 is a critical maturation and differentiation signal, and the absence of IL-15 results in paucity of *i*NKT cells in both the thymus and peripheral tissues^7^,^20^,^39^. Thus, the reduced thymic *i*NKT cell numbers in sγcTg is in line with impaired IL-15 signaling by sγc and the requirement for IL-15 in *i*NKT cell generation.

Importantly, thymic *i*NKT cells comprise a functionally and phenotypically heterogeneous population that contains distinct subsets of *i*NKT cells with differing degree of IL-15 dependency^6^,^37^. NKT1 cells, which correspond largely to stage 3 *i*NKT cells, express high levels of T-bet which in turn is critical for their maturation, survival and effector function^53^,^54^. Both NKT1 lineage choice and T-bet upregulation depend on IL-15 signaling^6^,^55^, so that impaired IL-15 signaling mostly affects NKT1 cells. NKT17 cells, on the other hand, depend exclusively on IL-7, but not IL-15, for their survival and homeostasis^56^. Thus, it was curious that sγc overexpression not only reduced number and frequency of IL-15-dependent NKT1 cells, but also of NKT17 and even NKT2 cells. However, these results can be reconciled when taking into account that sγc does only not inhibit IL-15 signaling, but also signaling by IL-2, IL-7, and presumably other γc cytokines^24^. Accordingly, sγc would not only block generation of IL-15-dependent NKT1 cells, but could also impair IL-7-dependent NKT17 cell development in the thymus. Because NKT2 cells were also reduced by sγc overexpression, this scenario further suggests a role of γc signaling in NKT2 lineage differentiation too.

Finally, the current results do not exclude the possibility that sγcTg could have interfered with cell proliferation to diminish thymic *i*NKT cell numbers. Positively selected stage 0 *i*NKT cells undergo massive (~100 fold) expansion upon differentiation into stage 1 *i*NKT cells which is dependent on c-Myc^57^. What cellular signals drive the proliferation is not clear, and we cannot formally discard the possibility that IL-15 could be involved in c-Myc-dependent proliferation during stage 0/1 transition. Whether this is indeed the case still remains to be tested. In sum, the inhibitory effect of sγc on IL-15 signaling *in vivo* and the impaired generation of thymic *i*NKT cells in sγcTg mice put forward a model of cytokine regulatory mechanism that requires integration of a role of sγc in controlling γc cytokine signaling.

## Materials and Methods

### Mice

C57BL/6 (CD45.2), CD45.1 congenic mice, and RAG^−/−^ mice were obtained from Charles River, Wilmington, MA, and from the Orient Bio, Korea. Soluble γc-transgenic mice were described and maintained in our colony^24^. Animal experiments were approved by the Pusan National University Institutional Animal Care and Use Committee (PNU-2014–0620) and the NCI Animal Care and Use Committee. All mice were cared for in accordance with Pusan National University School of Medicine and NIH guidelines.

### Flow cytometry

Single cell suspensions were prepared from the thymus of indicated mice. Data were acquired using LSR Fortessa or LSRII flow cytometers (BD Biosciences) and analyzed using FlowJo. Live cells were gated by forward scatter exclusion of dead cells stained with propidium iodide. The following antibodies were used for staining: TCRβ (H57–597), HSA (30-F1), IL-7Rα (A7R34), NK1.1 (PK136), IL-2Rα (PC61.5), IL-2Rβ (TM-β1), IL-4Rα (M1), Foxp3 (FJK-16s), RORγt (AKFJS-9) and isotype control antibodies, all from eBioscience; TCRγδ (GL3), CD44 (IM7), γc (4G3), CD4 (GK1.5 and RM4.5), and CD8α (53-6-7) from BD Biosciences; IL-9Rα (RM9A4), Bcl-2 (BCL/10C4), PLZF (9E12), and IL-21R (4A9) from BioLegend. Fluorochome-conjugated CD1d tetramers loaded with PBS-567 and unloaded controls were obtained from the NIH tetramer facility (Emory University, Atlanta, GA). Intranuclear Foxp3, PLZF, and RORγt proteins were detected using a Foxp3 staining kit according to the manufacturer’s instructions (eBioscience). Active caspase-3 induction was determined using the CaspGLOW™ fluorescein active caspase-3 staining kit (eBioscience).

### Quantitative Real-Time PCR

Total RNA was isolated from sorted thymocytes with the RNeasy Mini kit (Qiagen). RNA was reverse transcribed into cDNA by oligo (dT) priming with the QuantiTect Reverse transcription kit (Qiagen). Quantitative RT-PCR (qRT-PCR) was performed with an ABI PRISM 7900HT Sequence Detection System and the QuantiTect SYBR Green detection system (Qiagen). Primers sequences are as follows. sγc (F: 5′-CATGAACCTAGATTCTCCCTGCC-3′; R: 5′-TGATGGGGGGAATTGGAGIIIIICCTCTACA-3′) and *Rpl13* (F: 5′-CGAGGCATGCTGCCCCACAA-3′; R: 5′-AGCAGGGACCACCATCCGCT-3′). Gene expression values were normalized to those of *Rpl13* in the same sample.

### Expression of recombinant soluble γc protein

Recombinant sγc proteins were produced by transient transfection of 293 T human embryonic kidney cells with a mammalian expression vector pEGFP-N1 (Clontech) encoding a murine sγc cDNA. Cells were transfected with Lipofectamine™ 2000 (Invitrogen). Culture supernatant containing sγc proteins was collected 3 days after transfection and analyzed by Western blot for recovery and purity. Concentration of sγc protein was measured by ELISA as previously described^24^.

### *In vitro* stimulation with recombinant IL-15

Thymocytes were incubated *in vitro* with 20 ng/ml recombinant human IL-15 (Peprotech) in the presence or absence of recombinant sγc (500 ng/ml). Thymocytes were harvested 3 days after incubation, and stained for intracellular Bcl-2 expression. Annexin V staining was performed according to the manufacturer’s instructions (BD Biosciences).

### DN thymocyte subsets analysis

For DN1-DN4 thymocyte analysis, whole thymocytes were first incubated with the following biotinylated antibodies; anti-TCRβ, -B220, -CD8β, -GL3, -DX5, -MAC1, and -GR1, followed by FITC-conjugated streptavidin. FITC-signal negative thymocytes were considered as lineage marker negative cells (Lin^−^) and assessed for CD44 and CD25 expression using APC-conjugated anti-CD44 and PE-conjugated anti-CD25 antibodies (all from BD Biosciences). Intracellular Ki-67 staining of DN subsets was performed after fixation and permeabilization (Foxp3 transcription factor staining buffer set, eBioscience) of surface-stained thymocytes using anti-Ki-67 antibodies (eBioscience).

### Bone marrow chimeras

Radiation bone marrow chimeras were constructed by reconstituting lethally irradiated (600 Rad) RAG^−/−^ host mice with a total of 15 × 10^6^ T cell-depleted bone marrow (BM) cells either from WT (CD45.1) or sγcTg (CD45.2). For unequal bone marrow reconstitution, T cell-depleted BM cells from WT and sγcTg mice were mixed at 1:2 ratio (WT:sγcTg), and 15 × 10^6^ mixed BM cells were injected into irradiated RAG^−/−^ host mice. Chimeric mice were analyzed 8 weeks after reconstitution. Thymocytes from both BM chimeric mice were gated on CD45.1 or CD45.2 to distinguish WT and sγcTg donor cells.

### Statistical analysis

Data are shown as mean ± SEM. Statistical differences were analyzed by unpaired two-tailed Student’s *t*-test. P values of less than 0.05 were considered significant. *p < 0.05, **p < 0.01, ***p < 0.001. All statistical analysis was performed using GraphPad Prism.

## Additional Information

**How to cite this article**: Park, J.-Y. *et al*. Soluble γc cytokine receptor suppresses IL-15 signaling and impairs *i*NKT cell development in the thymus. *Sci. Rep.* **6**, 36962; doi: 10.1038/srep36962 (2016).

**Publisher’s note:** Springer Nature remains neutral with regard to jurisdictional claims in published maps and institutional affiliations.
